# Supplementary material for: Preliminary Evidence of Good Safety Profile and Outcomes of Early Treatment with Tixagevimab/Cilgavimab Compared to Previously Employed Monoclonal Antibodies for COVID-19 in Immunocompromised Patients
Source: Biomedicines. 2023 May 26;11(6):1540. doi: 10.3390/biomedicines11061540 (PMC10295521; doi:10.3390/biomedicines11061540)
Supplement: Supplementary file 1 [file biomedicines-11-01540-s001.zip › biomedicines-2197295-supplementary.pdf]

Supplementary Table S1. Signs and symptoms displayed by enrolled patients according to the treatment received.

**COVID-19 signs & symptoms**	**Overall**, N = 108	**mAbs**, N = 89	**TIX/CIL**, N = 19	**p-value**
Fever	60 (56%)	54 (61%)	6 (32%)	0.021
Cough	49 (45%)	40 (45%)	9 (47%)	0.847
Ageusia-dysgeusia	2 (1.9%)	2 (2.2%)	0 (0%)	>0.999
Anosmia	4 (3.7%)	3 (3.4%)	1 (5.3%)	0.544
Pharyngodynia	38 (35%)	29 (33%)	9 (47%)	0.221
Asthenia	27 (25%)	20 (22%)	7 (37%)	0.243
Headache	27 (25%)	22 (25%)	5 (26%)	>0.999
Myalgia	23 (21%)	19 (21%)	4 (21%)	>0.999
GI	11 (10%)	7 (7.9%)	4 (21%)	0.101
Dyspnea - tachypnea	2 (1.9%)	2 (2.2%)	0 (0%)	>0.999

GI: gastrointestinal tract

Supplementary Table S2. Monoclonal antibodies administered and doses of vaccines received among the enrolled patients.

**COVID-19 signs & symptoms**	**Overall**, N = 108	**mAb**, N = 89	**TIX/CIL**, N = 19	**p-value**
mAbs administered				<0.001
Tixagevimab/cilgavimab	19 (18%)	0 (0%)	19 (100%)	
Casirivimab/imdevimab	6 (5.6%)	6 (6.7%)	0 (0%)	
Bamlanivimab/etesevimab	15 (14%)	15 (17%)	0 (0%)	
Sotrovimab	68 (63%)	68 (76%)	0 (0%)	
**Vaccine dose(s)**				<0.001
0	6 (5.6%)	6 (6.8%)	0 (0%)	
1	3 (2.8%)	2 (2.3%)	1 (5.3%)	
2	25 (23%)	25 (28%)	0 (0%)	
3	62 (58%)	55 (62%)	7 (37%)	
4	11 (10%)	0 (0%)	11 (58%)	
Unknown	1	1	0	
